# Adoptive Immunotherapy Strategies with Cytokine-Induced Killer (CIK) Cells in the Treatment of Hematological Malignancies

**DOI:** 10.3390/ijms150814632

**Published:** 2014-08-21

**Authors:** Frederic Carsten Schmeel, Leonard Christopher Schmeel, Sanna-Marie Gast, Ingo G. H. Schmidt-Wolf

**Affiliations:** Medizinische Klinik und Poliklinik III, Center for Integrated Oncology (CIO), University Hospital Bonn, Rheinische Friedrich-Wilhelms-Universität Bonn, Sigmund-Freud-Straße 25, 53105 Bonn, Germany; E-Mails: cschmeel@uni-bonn.de (F.C.S.); schmeel@uni-bonn.de (L.C.S.); sun1979@hotmail.com (S.-M.G.)

**Keywords:** CIK (cytokine-induced killer) cells, clinical trials, immunotherapy, cancer treatment

## Abstract

Cytokine-induced killer (CIK) cells are a heterogeneous population of immune effector cells that feature a mixed T- and Natural killer (NK) cell-like phenotype in their terminally-differentiated CD3^+^CD56^+^ subset. The easy availability, high proliferation rate and widely major histocompatibility complex (MHC)-unrestricted antitumor activity of CIK cells contribute to their particularly advantageous profile, making them an attractive approach for adoptive immunotherapy. CIK cells have shown considerable cytotoxicity against both solid tumors and hematological malignancies *in vitro* and in animal studies. Recently, initial clinical experiences demonstrated the feasibility and efficacy of CIK cell immunotherapy in cancer patients, even at advanced disease stages. Likewise, the clinical application of CIK cells in combination with standard therapeutic procedures revealed synergistic antitumor effects. In this report, we will focus our consideration on CIK cells in the treatment of hematological malignancies. We will give insight into the latest advances and future perspectives and outline the most prominent results obtained in 17 clinical studies. Overall, CIK cells demonstrated a crucial impact on the treatment of patients with hematological malignancies, as evidenced by complete remissions, prolonged survival durations and improved quality of life. However, up to now, the optimal application schedule eventually favoring their integration into clinical practice has still to be developed.

## 1. Introduction

Over the last few decades, considerable progress has been made in the treatment of hematologic malignancies. However, the long-term efficacy remains disappointing, since most patients might eventually relapse and suffer from severe side effects caused by chemotherapy and radiation. Especially in elderly patients, which are most susceptible for hematologic malignancies, treatment-related adverse events are a major cause of drug discontinuation and reduced dose-intensity, thus aggravating treatment efficacy [1]. Therefore, additional and better-tolerated therapeutic strategies must be sought.

In recent years, adoptive immunotherapy has emerged as an innovative strategy in cancer treatment and has increasingly attracted attention as an adjuvant or even as an alternative therapy option. It aims at stimulating the patients’ immune system in order to trigger an antitumor immune response, eventually enabling the body’s natural abilities to better recognize and, finally, kill cancer cells. Among various immunotherapeutic approaches, in particular the adoptive cellular immunotherapy using cytokine-induced killer (CIK) cells has turned out to be a promising pathway. The first studies in the literature already demonstrated the high cytotoxic potential of CIK cells against transformed hematopoietic cells in *in vitro* and animal tumor models [2,3]. Very recently, an increasing number of clinical trials have reported that the adoptive CIK cell transfer revealed considerable antitumor efficacy and led to both significantly improved progression-free and overall survival (OS) in patients bearing different, especially solid, types of cancer, while being without serious side effects and well tolerated by the patients. Moreover, CIK cell transfusions were shown to positively influence the quality of life (QOL) and immune parameters of cancer patients known to present with impaired immune functions at advanced disease stages [4,5,6,7,8].

CIK cells meet decisive requirements to be effective in an immunotherapeutic approach. These cytotoxic CD8^+^ T-cells, also known as natural killer (NK) cell-like T lymphocytes, expand more rapidly and exhibit a stronger anti-tumor activity than other reported immune effector cells [3,9]. They are generated by the sequential *ex vivo* incubation of human peripheral blood mononuclear cells (PBMC) with interferon-gamma (IFN-γ), anti-CD3 antibody and recombinant human interleukin (IL)-2 [2].

After this expansion procedure, two predominant subsets of either CD56-positive or CD56-negative CIK cells can be distinguished within the heterogeneous, mainly CD3^+^ T-cell culture, whereby the exact proportions of either cell type may vary dependent on the application scheme used. Among them, the terminally-differentiated CD3^+^CD56^+^ subset represents the cell type with the highest tumor killing abilities. This subset acquired, as a key feature, a double T-cell and NK cell phenotype and, thus, exerts a potent and widely MHC-unrestricted anti-tumor cytotoxicity against a broad range of cancer cells [3,10]. Interestingly, these CD3^+^CD56^+^ cells do not derive from NK cells, but develop from proliferating CD3^+^CD56^−^ T-cells, which are still hampered by residual alloreactivity across Human Leukocyte Antigen (HLA) borders [3,11,12].

CIK cells’ anti-tumor activity is perforin mediated and was mainly attributed to the cell-cell contact-dependent natural killer group 2 member D (NKG2D) cell-surface receptor, since antibody blocking experiments using anti-NKG2D antibody or siRNA showed that CIK cells mainly lost their T-cell receptor (TCR)-independent antitumor cytotoxicity against malignant cells. Most CIK cells express NKG2D, and its activity is associated with the adaptor molecule DNAX-activating protein of 10 kDa (DAP10), which is, in turn, upregulated in CIK cells at high doses of IL-2 and not restricted to the CD3^+^CD56^+^ fraction [13]. Both solid and hematologic tumor cells overexpress NKG2D ligands, typically MHC class I chain-related molecules (MIC) A/B and members of the UL16 binding proteins (ULBP) family, making them a favorable target of CIK cell-mediated cytolysis [14,15,16]. CIK cells also express some other activating NK receptors, like DNAM-1, NKp30, NKp44 and NKp46, which have been suggested to influence tumor recognition, as well; however, little is currently known about their involvement in the antitumor activity of CIK cells. Along with that, terminally-differentiated CIK cells are characterized by the expression of CD45RA^+^, CCr7^−^, CD11a^+^ CD62L^−/+^, CD27^+^ and CD28^−^ with more late effector features present in the CD3^+^CD56^+^ subset than in CD56^−^ cells [11,17].

Many adoptive immunotherapies using various killer cells, *i.e.*, standard lymphokine-activated killer (LAK) cells and tumor-infiltrating lymphocytes (TILs), have merely shown limited efficacy in clinical trials, due to their poor expansion ability and low antitumor activity [18]. Particularly, LAK cells, after expansion from peripheral PBMCs at high doses of IL-2, revealed an MHC-unrestricted cytotoxicity against tumor cells, as CIK cells do. In comparison, however, CIK cells can be obtained more efficiently and turned out to be more specific in the killing of tumor cells [9]. Thus, CIK cells, due to their advantageous cytotoxic profile and recent clinical results, might have the potential to substantially enhance future cancer immunotherapy. This is why it took no long for numerous clinical studies to investigate CIK cells’ efficacy towards a broad scope of neoplasms. In this report, we will give insight into the efficacy of CIK cells with a focus on hematological malignancies. Therefore, we will summarize the latest advances in CIK cell immunotherapy and outline the most prominent results obtained in clinical studies that utilized CIK cells for the therapy of hematological neoplasms.

## 2. Improving CIK (Cytokine-Induced Killer) Cell Proliferation and Efficacy

In 1991, Schmidt-Wolf *et al.* [2] developed a standard protocol for the generation and expansion of CIK cells, which our workgroup still uses until today. Accordingly, CIK cells can be generated *in vitro* by the addition of IL-2 to PBMCs. Nevertheless, by now, CIK cell cultivation conditions have been extensively modified, and much research is ongoing to improve both the propagation and tumor-specific cytotoxicity. Particularly, the use of cytokines other than IL-2 has been addressed so far. A further special focus was put on the suppression of T-regulatory cells (Tregs) within the CIK cell culture, since their removal led to enhanced cytotoxicity [19].

Lin *et al.* [20] found out that the use of IL-6 every two to three days led to the increased proliferation rate and cytotoxic activity of CIK cells. This was broadly in line with the observation that the proportion of the desired CD3^+^CD56^+^ effector cells was significantly increased, eventually resulting in a higher *in vitro* cytotoxicity. Simultaneously, the proportion of Tregs within the CIK cell culture was also significantly decreased, suggesting that IL-6 might additionally inhibit the immunosuppressive functions of Tregs.

Recently, Heninger *et al.* [21] reported on similar results showing that the cultivation with IL-7 also leads to a higher proliferation rate and cytotoxicity as compared to solely IL-2 expanded CIK cells. Interestingly, this observation was accompanied by a highly abrogated immunosuppressive Tregs function. IL-7 gene-transfected CIK cells provided these beneficial features as well [22].

With particular regard to hematological malignancies, likewise, the alternative use of IL-15 instead of IL-2 revealed considerable advantages. CIK cells stimulated by IL-15 exhibited an enhanced cytotoxic activity against acute lymphoblastic leukemia and lymphoma cell lines, as well as against primary acute myeloid and defined lymphoblastic leukemia cells [23]. Additionally, the percentages of CD3^+^CD56^+^ cells significantly increased, while the immunosuppressive functions of Tregs and IL-35 were also depressed [24].

Furthermore, the cultivation with IL-21 was shown to increase the expression of IL-21 receptors, perforin, granzyme B, Fas ligand, IFN-γ and tumor necrosis factor-α. Besides that, the relative proportions of the desired CD3^+^CD56^+^ cell population increased, but without enhancing the general proliferation rate [25]. Based on these qualities, IL-21 might become another interesting candidate for future CIK cell cultivation.

Finally, there is growing evidence that co-cultivation with dendritic cells (DC) could additionally improve the anti-tumor toxicity and reduce the number of Tregs within the CIK cell culture. Moreover, the combined cultivation (DC-CIK) was capable of improving the expansion and frequency of CD3^+^CD56^+^ cells in the amplified population, while the interactions between DCs and CIK cells led to an increase of the IL-12 secretion [26,27,28,29,30].

## 3. Alloreactivity

Within the framework of hematological malignancies, CIK cells’ alloreactivity is of major importance, since allogeneic hematopoietic stem cell transplantation (HSCT) often remains as the only curative therapeutic option for many patients affected by hematologic neoplasms. A major benefit of allogeneic HSCT in the treatment is the graft-*versus*-tumor (GVT) effect conferred by lymphocytes contained within the graft. This GVT effect can even be utilized as a therapeutic means after the relapse of disease by the infusion of donor lymphocytes (donor lymphocyte infusion (DLI)). However, these lymphocytes can also be responsible for the potentially severe complication of graft-*versus*-host disease (GVHD). Importantly, in this respect, CIK cells displayed reduced alloreactivity across HLA barriers. In a murine model, CIK cells exhibited strong *in vivo* antitumor toxicity by GVT activity with minimal to no GVHD after transplantation across MHC barriers into tumor-bearing hosts [31]. Another murine model of the same group revealed the association of high IFN-gamma levels produced by CIK cells with the reduction of GVH activity; cells expanded from IFN-gamma knock-out animals caused GVHD [32]. 

Most interestingly, two distinct subsets of CIK cells were shown to be responsible for either alloreactivity or anti-tumor activity. Antitumor efficiency was mainly caused by CD3^+^CD56^+^ cells, whereas alloreactivity was restricted to the CD3^+^CD56^−^ fraction. This might indicate that a depletion of CD3^+^CD56^−^ cells can result in a reduced risk of GVHD without alleviated tumor-killing capacity and, thereby, opens up safer treatment modalities with CIK cells, even across major HLA barriers [12].

## 4. Phase I Clinical Studies on Autologous CIK Cells

The very first clinical report on the administration of CIK cells for the treatment of various malignancies was performed by Schmidt-Wolf *et al.*, 1999 [10]. Here, a 54 year-old man, suffering from follicular lymphoma grade I, received a total of 1.84 × 10^9^ autologous CIK cells in two cycles. There was no treatment toxicity, and a pre-existing bone marrow involvement as the only sign of disease resolved after CIK cell therapy. This was scored as a complete clinical remission (CR). Along with this clinically-improved presentation, the patient was shown to have increased serum levels of IFN-γ and transforming growth factor beta (TGF-β) after CIK cell treatment. Leemhuis *et al.* [33] subsequently reported on a phase I study for the treatment of relapsed lymphomas after autologous stem cell transplantation (HSCT). Therefore, they enrolled nine patients with an age range from 23 to 67 years. Seven patients presented with advanced Hodgkin’s disease (HD) and two patients with B-cell non-Hodgkin’s lymphoma (NHL). A total of 21 infusions were given to nine patients. The number of CIK cells infused ranged from 1.0 × 10^9^ to 1.0 × 10^1^^0^ per treatment. The toxicity was minimal, and there were no immediate side effects to the infusions. Three patients with HD and one patient with NHL responded to the therapy. Two patients with HD achieved partial remissions (PR), and two patients had stabilization of disease (SD); one for more than 18 months [33].

Besides patients bearing solid tumors, Olioso *et al.* [34] enrolled six patients suffering from advanced lymphomas (three HD) in a pilot phase I trial. The median number of transferred cells per patient was 7 × 10^9^ (range 2.2–21). CIK cells were administered every three weeks three times. Merely one patient with centroblastic-centrocytic NHL achieved and sustained CR, even after 44 months of follow up. The remaining patients did not clinically respond to CIK cells at all. Only mild fever was observed in the patients collectively. Interestingly, immunological effects, such as significantly increased levels of IFN-γ and TNF-α, were solely found in responding patients. In contrast, non-responding patients showed no significant alterations in cytokine levels [34].

Yang *et al.* [35] infused 2–3 × 10^9^ autologous CIK cells into nine elderly patients with B-cell NHL, followed by subcutaneous injection of IL-2 at a single daily dose of 1 × 10^4^ U/day for 10 consecutive days. In seven and two patients, eight and four of these cycles were completed, respectively. The corresponding CIK cell treatment cycles were repeated every four weeks. All nine patients did not develop any adverse reactions. On the contrary, lymphoma symptoms relieved, and the quality of life was obviously improved. Additionally, the relative numbers of CD3^+^, CD3^+^CD8^+^ and CD3^+^CD56^+^ T-cells increased significantly, and unspecific tumor marker levels, like β2-microglobulin and lactate dehydrogenase (LDH), decreased. Overall, the results were promising, since eight of nine patients achieved CR [35].

The same treatment procedure and schedule was applied by Liu *et al.* [36] to evaluate the effectiveness of CIK cell treatment combined with IL-2 in the treatment of six geriatric patients with myelodysplastic syndrome (MDS). Again, no severe adverse effects occurred. Along with CIK cell treatment, inflammation frequency and high fever durations were significantly reduced. Likewise, as stated by the authors, a reduction in blood transfusions required to stabilize hemoglobin levels could be observed during the stable stage of disease. In this context, patients did not receive erythropoietin-stimulating agents. Again, the percentages of CD3^+^, CD3^+^CD8^+^ and CD3^+^CD56^+^ T-cells increased significantly, and patients experienced an improved QOL; however, transformation from myelodysplastic syndrome to high-risk subtypes could not be interrupted [36].

As the next step, Yang *et al.* [37] enrolled 20 patients in a phase I trial. The patients’ age ranged from 57–93 years with a median age of 83 years. Among them, nine were diagnosed with lymphoma, five with MDS, two with multiple myeloma (MM), three with chronic lymphocytic leukemia (CLL) and one with acute myeloid leukemia (AML). Once more, Yang *et al.* [37] combined the CIK cell infusions (2–3 × 10^9^) with subcutaneous injections of IL-2 (1 mU/day) for 10 consecutive days after each infusion. Six patients received four cycles, and the remaining 14 were administered eight cycles. Three patients developed malaise and low-grade fever, which disappeared after symptomatic treatment. Positive immunologic effects were observed as evidenced by an increased number of CD3^+^, CD3^+^CD8^+^ and CD3^+^CD56^+^ cells. Moreover, the levels of serum β2 microglobulin and LDH were markedly decreased. After follow up, CR, PR and SD were observed in eleven, seven and two patients, respectively. In detail, five of nine lymphoma patients experienced an improved clinical state with even three patients improving from progressive disease to CR, while four patients could maintain their previous disease state as determined prior to CIK cell treatment; the disease state of all remaining eleven patients improved, with nine of them even achieving CR or PR. Additionally, the QOL markedly improved as demonstrated by an increased Karnofsky score from 57% (40%–90%) to 83% (60%–100%) [37]. Subsequently the same group reported on nine geriatric patients (aged from 65–90 years) with diffuse large B-cell lymphoma, which were pretreated with four courses of R-CHOP (Rituximab, Cyclophosphamide, Doxorubicin Hydrochloride (Hydroxydaunomycin), Vincristine Sulfate (Oncovin) and Prednisone) before CIK cell treatment. After pretreatment, seven patients achieved PR, and two achieved CR. The patients received eight cycles, as described in the workgroup’s previous study. A total of 5 × 10^9^–1 × 10^10^ CIK cells were given per infusion. Two patients developed mild fatigue and low-grade fever. At the study endpoint, CRs were observed in all patients, and lymphoma-related symptoms were markedly improved with a survival time of 24–35 months. Again, increased proportions of CD3^+^, CD3^+^CD8^+^ and CD3^+^CD56^+^ cells were observed [38].

By applying almost the same therapy protocol, the workgroup then assessed the efficiency of CIK cells towards B-cell CLL. This time, opposite to their prior trials, thymic peptide α1, used for treating diseases associated with immune dysfunction, including viral infections, such as hepatitis B and C, was given subcutaneously as an immunoenhancement at a dose of 1.6 mg/day for 14 days before and after CIK cell administration. Forty six cycles of CIK cell infusions (each (4–6) × 10^9^ CIK cells) plus IL-2 were completed in five CLL patients. Besides the interesting observation that the CIK cell amplification time, the cell amount and antitumor cytotoxicity was significantly increased after thymic peptide α1 treatment, patients’ clinical state improved as evidenced by transformation from PR to CR in three patients, from SD to PR in one patient and from progression of disease to SD in one patient [39].

Very recently, the group of Yang *et al.* [40] reported on a 68 year-old man with advanced refractory multiple solitary plasmacytomas and multiple bone lesions. CIK cells were infused once a month (2–8 × 10^9^ cells), and 21 courses of CIK cell treatment had been administered. All bone lesions were relieved and the patient achieved CR, lasting till the end of follow-up [40].

Merely one phase I study reported on the non-superior anti-tumor efficiency of autologous CIK cell therapy. In five elderly patients with acute myeloid leukemia (AML), no instances of side effects were observed, and immunotherapy could significantly reduce both the incidence of infections and high fever durations. Without improving the clinical disease state, but worthy of being mentioned, CIK cell treatment could significantly reduce the volume of erythrocyte infusions required to maintain hemoglobin levels as compared with the patients’ previous transfusion needs. However, even if CIK cell treatment could control the patients’ general condition, the outcome of AML could not be changed [41].

## 5. Phase I Clinical Studies on Allogeneic CIK Cells

The first promising results obtained by the utilization of CIK cells in patients that had relapsed after allogeneic HSCT were reported by Introna *et al.*, 2007 [42]. They enrolled eleven patients with a median age of 53 (range 24–62), of whom four presented with AML, three with HD, one with pre-B acute lymphoblastic leukemia (pre-B ALL), one with chronic myelomonocytic leukemia (CMML) and two with MDS. Six and five of them had undergone sibling or unrelated donor HSCT, respectively. With the exception of two patients, they were pretreated with reduced intensity protocols prior to HSCT. After a median time of 315 days from transplantation, patients were given between one to seven infusions (median 2) at three to four week intervals with a median number of 12.4 × 10^6^ (7.2–87.4 × 10^6^) CIK cells. Despite the information that all transfusions were well tolerated, as there were no immediate or late adverse reactions, four patients developed acute graft-*versus*-host disease (GVHD) within 30 days after the last infusion. Two of them progressed into extensive chronic GVHD (grade II). However, these GVHD cases only occurred in patients that received donor lymphocyte infusions (DLI) before the treatment with CIK cells. Consequently, this might hamper certain conclusions on the induction of GVHD by allogeneic CIK cells. Six patients did not benefit from CIK cells and eventually died. It is, however, noteworthy that one patient experienced SD, one PR and three even achieved CR [42].

In 2012, Laport *et al.* [43] reported on a comparable approach with 18 patients who relapsed after allogeneic HSCT using a matched sibling donor. The enrolled patients collectively had a median age of 53 years and consisted of patients with the following diagnoses: NHL (*n* = 5), AML (*n* = 3), MM (*n* = 3), CLL (*n* = 2), ALL (*n* = 2), MDS (*n* = 2) and HD (*n* = 1). Twelve of them had received a reduced intensity conditioning regimen, and six had received myeloablative regimens. All but two patients were treated with cytoreductive therapy, including chemotherapy, corticosteroids, surgical resection or DLI, before receiving CIK cells. After a median time of four months (range 1–34 months) from relapse to infusion, patients were given merely one infusion of CIK cells at escalating doses of 1 × 10^7^ (*n* = 4), 5 × 10^7^ (*n* = 6) and 1 × 10^8^ (*n* = 8) cells/kg. Acute GVHD, grades I–II, and limited chronic GVHD were developed by two and one patient, respectively. The median overall survival was 28 months and the median event-free survival was four months. Eight patients died due to relapsed disease within the follow-up duration of 1–69 (median 20) months [43]. However, longer remissions were seen in five patients after CIK cell administration compared to remission durations from allogeneic transplantation to relapse.

In another study, the feasibility and toxicity of cord blood-derived CIK cells were under investigation. Therefore, five patients with relapsed acute leukemia after cord blood transplantation were treated with HLA-matched cord blood-derived CIK cells. No acute or delayed adverse events were observed. A partial response concomitantly with the development of acute grade III GVHD was seen in one patient [44].

Linn *et al.* [45] provided some interesting results suggesting the efficiency of allogeneic CIK cells in patients that did not respond to various individualized chemotherapy regimens and donor lymphocyte infusion (DLI). Allogeneic CIK cells were successfully generated for a total of 24 patients, of whom, eight were diagnosed with AML, three with ALL, three with HD, one with CML and one with NHL. A total of 55 infusions were done for 16 patients with doses ranging from ten to 200 million CD3^+^ cells/kg; prior to the infusions, eight patients were excluded from the study. Acute GVHD occurred in three, but discontinued after treatment. For six patients, CIK cell therapy was not beneficial. The response of five patients was not assessed due to concomitant use of other agents that might have influenced the observed clinical result. Eventually, it was stated that the results of two ALL, two HD and one AML patient were suggestive of the clinical efficacy of CIK cells [45].

## 6. Phase I/II Clinical Studies on Autologous CIK Cells

Jiang *et al.* [46] evaluated the efficacy of CIK cell therapy followed by chemotherapy in 41 patients with acute leukemia. Nineteen patients received CIK cell treatment prior to chemotherapy, and 22 patients received chemotherapy only, thereby constituting the control group. The programs of chemotherapy in the two groups were similar. In the immunotherapy group, eight patients received four courses, two patients three courses, five patients two courses and four patients merely one course of CIK cell treatment. The amount of transfused CIK cells per patient were (14.2 ± 8.5) × 10^9^/L. All patients achieved and maintained CR for more than six months after chemotherapy. After for years of follow up, the continuous CR rate was 73.4% in the CIK group *vs.* 27.3% in the control. Thus, the CR rate was significantly higher in patients receiving CIK cells before chemotherapy than in patients who received chemotherapy only. Moreover, the efficacy of ≥3 courses was better than in patients who received <3 courses of treatment. CRs lasted until the end of follow-up in the combination group. However, four of nine patients who received <3 courses of CIK cell treatment relapsed after CR during the follow-up duration [46].

During another study, thirteen AML patients in remission after autologous HSCT and eleven patients with CML on imatinib with residual disease detectable by polymerase chain reaction (PCR) received CIK infusions at three weekly intervals. Out of thirteen AML patients and ten CML patients, eleven and ten successfully generated autologous CIK cells, respectively. Three, one, five and two AML patients were given one, two, three and four infusions, respectively. These infusions were administered after a median time of 16 days post-transplant (range 13–37 days) when patients had recovered from cytopenia after HSCT. In the CML group, all patients received four infusions. Each infusion contained a median cell dose of 12.72 × 10^9^ (1.3–78.6 × 10^9^) cells for the AML group and 25.72 × 10^9^ (13.76–54.94 × 10^9^) cells for the CML group. Fever occurred as the only side effect and was easily controllable. At the end, no clinical benefit was observed in the AML group, as the relapse-free survival time was not significantly different compared to historical controls. Likewise, no benefit was seen in the CML group [47].

The only clinical study that combined CIK cells with dendritic cells (DC) for the treatment of multiple myeloma was reported by Zhong *et al.*, 2012 [48]. Sixty patients were randomly divided into two groups. One patient group built the control group and received chemotherapy only; the second group was treated with chemotherapy plus DC-CIK. DCs were cultured *in vitro* by treating PBMCs with granulocyte-macrophage colony-stimulating factor (GM-CSF) and IL-4 and subsequently pulsed with MM cell lysate. Both CIK cells and DCs were adoptively transferred with 2.5–5 × 10^9^ cells per transfusion. After immunotherapy, the percentages of CD3, CD8 and CD56 cells were significantly increased. In addition, the levels of HSP70 and the Th1/Th2 ratio were higher, while the proportions of anti-inflammatory TGF-beta, β2-microglobulin and CD4^+^CD25^+^ regulatory T-cells decreased. At the study endpoint, OS was significantly improved, with 42.9 *vs.* 33.6 months in the immunotherapy and chemotherapy group, respectively. Moreover, the QOL and clinical indices in the joint group were significantly better than in the chemotherapy group [48].

## 7. Overall Benefits and Toxicity

Taken together, 206 patients with an age ranging from 18–93 years (median 53 ± 17.76) were enrolled in 17 clinical studies. They received between one and 21 infusions with a mean and median number of 5.5 and 3.5 infusions, respectively. The CIK cell dose for each infusion ranged from 1 × 10^8^–2.8 × 10^10^ with a median number of 5 × 10^9^ CIK cells. Side effects were mostly minor, as shown in Table 1. However, mild fever (37.5–40 °C) often occurred after CIK cell infusions, but lasted only up to 24 h after each infusion and was easily treatable with symptomatic means, like non-steroidal antiphlogistic medication. In a single study, two patients developed transient ventricular arrhythmias. However, the etiology of the arrhythmias and a possible association with CIK cell therapy was never elucidated.

**Table 1:** Side reactions associated with CIK cell therapy. GVHD, graft-*versus*-host disease; CIK, cytokine-induced killer.

Side Reaction	Frequency in %
Fever (37.5–40 °C)	26.37
GVHD (grade I–II)	14
Ventricular arrhythmia	1
Chills	1
Fatigue	1

Side reactions following CIK cell immunotherapy in 206 patients.

CRs and PRs were observed in many patients that had undergone CIK cell therapy. Table 2 briefly summarizes the respective results and gives an overview on the described clinical trials.

**Table 2:** Clinical studies using CIK cells.

Study Reference	Phase	Cancer Disease	Patients (*n*)	Therapy	Clinical Response
Schmidt-Wolf *et al.* [10]	I	Colon carcinoma (7)	10	IL-2-gene transfected, autologous CIK cells	CR (1), SD (3); CIK cell therapy led to CR in one patient with follicular lymphoma
		follicular lymphoma (2)			
		Renal cell carcinoma (1)			
Leemhuis *et al.* [33]	I	HD (7)	9	autologous CIK cells	PR (2), SD (2)
		B-cell NHL (2)			
Olioso *et al.* [34]	I	HD (2)	12	autologous CIK cells	CR (3), PR (1), SD (2); one patient with B-cell NHL achieved PR; enhancement of immune functions, significantly prolonged OS in clinical responders
		B-cell NHL (4)			
		Renal cell carcinoma (5)			
		Hepatocellular carcinoma (1)			
Yang *et al.* [35]	I	B-cell NHL	9	autologous CIK cells + IL-2	CR (8), PR (1); enhancement of immune functions, improved quality of life
Liu *et al.* [36]	I	MDS	6	autologous CIK cells + IL-2	Transformation from MDS to high-risk subtypes could not be changed by CIK cell treatment, but inflammation frequency was significantly reduced
Yang *et al.* [37]	I	HD (1)	20	autologous CIK cells + IL-2	CR (11), PR (7), SD (2); CIK cell treatment significantly enhanced immune functions and quality of life
		B-cell NHL (8)			
		MDS (5)			
		MM (2)			
		CLL (3)			
		AML (1)			
Lu *et al.* [38]	I	B-cell NHL	9	autologous CIK cells	CR (9); the Karnofsky score was significantly higher
Cai *et al.* [39]	I	CLL	5	autologous CIK cells + TP α-1	CR (3), PR (2), SD (1)
Yang *et al.* [40]	I	MM	1	autologous CIK cells	CR (1) without disease progression for 40 months
Yang *et al.* [41]	I	AML	5	autologous CIK cells	No clinical response
Introna *et al.* [42]	I	AML (4)	11	allogeneic CIK cells	CR (3), PR (1), SD (1); GVHD in four patients
		HD (3)			
		MDS (2)			
		ALL (1)			
		CML (1)			
Laport *et al.* [43]	I	B-cell NHL (5)	18	allogeneic CIK cells	CR (5) greater than 1 year; GVHD in three patients. The median overall survival was 28 months and median event free survival was 4 months
		AML (3)			
		MM (3)			
		CLL (2)			
		ALL (2)			
		MDS (2)			
		HD (1)			
Introna *et al.* [44]	I	AML (4)	5	cord-blood derived, allogeneic CIK cells	PR (1) in one patient presenting with acute GVHD
		ALL (1)			
Linn *et al.* [45]	I	AML (8)	16	allogeneic CIK cells	Clinical response attributable to CIK cell infusion in five patients; acute GVHD in three patients
		ALL (3)			
		HD (3)			
		CML (1)			
		B-cell NHL (1)			
Jiang *et al.* [46]	I	Acute leukemia	19	autologous CIK cells, followed by chemotherapy	CR rate was 73.4% in the CIK group *versus* 27.3% in the control group
Linn *et al.* [47]	I/II	AML (11)	21	autologous CIK cells	No clinical response
		CML (10)			
Zhong *et al.* [48]	II	MM	30	autologous DC-CIK cells + chemotherapy	OS, quality of life and clinical indices were significantly higher than in chemotherapy group

ALL: acute lymphoid leukemia; AML: acute myeloid leukemia; CIK: cytokine-induced killer; CLL: chronic lymphoid leukemia; CML: chronic myeloid leukemia; CR: complete remission; DC: dendritic cell; GVHD: graft-*versus*-host disease; HD: Hodgkin’s disease; IL-2: interleukin-2; MDS: myelodysplastic syndrome; MM: multiple myeloma; NHL: non-Hodgkin’s lymphoma; OS: overall survival; PR: partial remission; SD: stable disease; TP α-1: thymic peptide alpha-1.

## 8. Conclusions

Major advances have been made in the field of adoptive immunotherapy, which has been considered a promising approach in taking advantage of the stimulated patient’s immune system to recognize and eventually eliminate tumor cells. Among them, particularly CIK cells emphasize these natural abilities of the human immune system to destroy cancer cells and, thus, represent a unique immunotherapeutic approach, which enriched our therapeutic repertoire in the struggle against cancer. CIK cells exhibited significant antitumor activity in preclinical experiments and animal tumor models [2,3]. Their technically simple and inexpensive cultivation and their non-MHC-restricted cytotoxicity strongly promoted the transition from preclinical experiments to clinical application. Hence, it did not take very long until CIK cells were utilized in clinical trials facing a wide range of different tumor entities. Many of these trials reported on valuable results, as CIK cell therapy improved the quality of life and prolonged the patients’ survival [5,6,7,30].

Here, in the context of hematological malignancies, we found corroborative evidence for the assumption that CIK cell immunotherapy benefits the patients in terms of recurrence prevention, improved quality of life and prolonged progression-free survival. Considerable antitumor effects were observed in various hematological malignancies, and most interestingly, side reactions were little or even absent.

The vast majority of patients that received autologous CIK cells experienced an improved disease state accompanied by an improved QOL and general presentation, even in advanced disease stages. 

Of 123 patients, five patients achieved SD, 18 patients achieved PR and even 41 patients achieved CR; a proportion of 52% achieved an improved disease stage with an overall response rate (ORR) of 48%. In many cases, immunological parameters improved, tumor markers decreased and inflammation frequency was less than before CIK cell therapy. Despite these promising numbers, however, there were some studies suggesting that CIK cell therapy might not have a substantial impact on the course of hematological neoplasms. Especially, AML patients were shown to benefit least. The authors of the respective studies explained that either improper timing of the infusions or an insufficient quantity of CIK cells might have hindered more positive results [41,47]. Besides these factors, Linn *et al.* [47] describe the lack in CD62L and CD27 expression of the CD3^+^CD56^+^ subset, which is critical for lymphoid homing properties and anti-tumor immunity, as another imaginable reason for the reduced long-term efficacy of CIK cells [47].

Most interestingly, CIK cell therapy was not associated with any severe adverse effects, and more frequent side effects were mild, well tolerated by the patients and easy to control. However, approximately one quarter of the whole patient collective, including allogeneic settings, developed mild fevers, which eventually resolved after 24 h or could be treated with anti-inflammatory treatment. Other rather rare adverse reactions were also mild and easily treatable.

Apart from the use of CIK cells in the autologous treatment line, the non-MHC-restricted cytotoxic profile and reduced alloreactivity renders them attractive for the application across major HLA barriers, especially after allogeneic HSCT. As in autologous studies, the four clinical studies utilizing allogeneic CIK cells could prove the feasibility and safety of CIK cell therapy, since adverse reactions were equally frequent and mild. The efficiency was less favorable, but also showed initial encouraging results. In this regard, the major emphasis of these trials should not merely be placed on the CIK cell potency, but on the observation that allogeneic CIK cells caused only little toxicity, particularly GVHD. Only ten of 50 patients developed GVHD, in most cases, concomitantly with a clinical response [42,43,44,45]. Of note, most of these 50 patients received DLI prior to CIK cell infusions, but showed no sufficient response. This underlines the preclinical observations that the use of CIK cells as an updated form of DLI might be superior in terms of reduced alloreactivity and disease control. However, up to now, the reliable clinical use of allogeneic CIK cells has been limited to combination therapies with standard therapeutic regimens and should be preferred in more indolent diseases rather than in acute leukemia.

The described clinical studies predominantly suggest a superiority of CIK cell therapy as a second-line treatment along or after standard therapeutic procedures. In fact, this assumption is probably due to the currently applied inclusion criteria and safety concerns. Nevertheless, building upon the great safety profile and the relatively harmless extraction of CIK cells, this interesting therapeutic approach could also benefit the patients as a first-line treatment modality when combined with common therapeutic strategies, especially in the autologous form. However, substantial conclusions on the potential efficiency and on the question to what extent CIK cell therapy might influence the survival durations cannot be drawn at the moment. It is actually not difficult to find an explanation for this deadlocked situation, since there exists a vast heterogeneity in terms of study design, numbers of infused CIK cells, different proportions of CIK cell phenotypes within the infusions and outcome assessment. Moreover, the proper timing of the infusions seems to be critical, as well.

To face these disparities and to gradually homogenize future trials, we decided to found the International Registry on CIK cells (IRCC) in 2010. Our primary aim is providing standard instruments for the design and the outcome assessment of future trials, but also data collection and subsequent analysis to present the latest state of CIK cell efficacy and to broaden the acceptance and interest in this promising treatment approach [4,5].

Ongoing research efforts are decisive to address these open questions. The most crucial issue will be the development of superior treatment protocols. Most interesting in this regard appears the reduced alloreactivity of CIK cells by the depletion of CD3^+^CD56^−^ cells. Taking appropriate measures to deplete these cells within the heterogeneous amplification product could possibly contribute to even safer CIK cells in allogeneic treatment lines. Especially innovations in the generation of CIK cells by applying other cytokines than IL-2 or cytokine gene-transfection might not only accelerate the propagation of CIK cells, but also improve their *in vivo* cytotoxicity. Another valuable approach is the co-cultivation with antigen-presenting DCs, as their interaction renders CIK cells more aggressive and speeds up the expansion of the desired CD3^+^CD56^+^ subset. Hence, the implementation of the DC-CIK strategy, as well as the selection of varying cytokines seems tempting. It is regrettable that, except for DC-CIK, currently, none of the abovementioned improvement strategies have found a route to clinical application for the treatment of hematological neoplasms. However, larger scaled clinical trials covering these new strategies are indispensable and will hopefully disclose the potential of CIK cells in combination with further established therapies. In conclusion, much effort has been made in recent years to optimize the efficacy of CIK cells and to realize the translation of CIK cells into clinical practice, where they proved safe and efficient. Plenty of innovative ideas will promote the progression in this fascinating field, and CIK cells might, therefore, play an outstanding role in the treatment of hematological malignancies in the future.
